# Do Sex and Gender Interact with the Biological Actions of Taurine? A Critical Rereading of the Literature

**DOI:** 10.3390/ijms26168097

**Published:** 2025-08-21

**Authors:** Giuseppe Seghieri, Ilaria Campesi, Giancarlo Tonolo, Federico Bennardini, Isabella Stendardi, Rosanna Matucci, Flavia Franconi

**Affiliations:** 1 Scienze Biomediche, Sperimentali e Cliniche, Università di Firenze, 50121 Firenze, Italy; giuseppe.seghieri@gmail.com; 2 Dipartimento di Scienze Biomediche, Università degli Studi di Sassari, 07100 Sassari, Italy; 3 Laboratorio Nazionale di Farmacologia e Medicina di Genere, Instituto Nazionale Biostrutture Biosistemi, 07100 Sassari, Italy; franconi.flavia@gmail.com; 4 Struttura Complessa di Diabetologia della Asl Gallura, 07026 Olbia, Italy; giancarlo.tonolo@aslgallura.it; 5 Dipartimento di Chimica e Farmacia, Università degli Studi di Sassari, 07100 Sassari, Italy; fbennardini@gmail.com; 6 Dipartimento di Neuroscienze, Psicologia, Area del Farmaco e Salute del Bambino, Università di Firenze, 50121 Firenze, Italy; i.stendardi@inwind.it (I.S.); rosanna.matucci@unifi.it (R.M.)

**Keywords:** taurine, cardiovascular diseases, diabetes mellitus, developmental programming, sex and gender differences

## Abstract

In humans, taurine (TAU) is a conditionally essential nutrient that exhibits pleiotropic activity in several and different biological processes suggesting its use in the prevention and therapy for a long time. However, its actual role in prevention and treatment is still incomplete and unclear. This review focuses on the potential therapeutic effect of TAU in genetic diseases, cardiovascular diseases (heart failure, hypertension), metabolic syndrome, and on the first pandemic of the third millennium, namely, diabetes mellitus and some gestational diseases such as gestational diabetes, intrauterine growth restriction, and pre-eclampsia, discussing the role of TAU in developmental trajectory. Previous preclinical and clinical TAU investigations predominately enrolled male animals, including humans, even though sex and gender differences play a critical role both in numerous physiological and pathological conditions. This review aims to outline some biological actions of TAU and evidences the sex and gender gap must be reduced in order to establish the role of TAU in prevention and therapy for all individuals.

## 1. Introduction

Taurine (TAU) is a sulfonate-beta-amino acid that weighs 125.15 g/mol and is characterized by an amino group and a sulfonic acid group attached to the beta carbon, exhibiting high water solubility and optical inactivity [1]. Additionally, TAU is not incorporated into proteins.

In humans, TAU is regarded as a semi-essential amino acid, though a specific dietary reference intake has not yet been established [1]. Nonetheless, it is generally considered that the Western diet provides a sufficient amount of TAU, with adult intake estimated to range from 40 to 400 mg per day [1]. TAU-rich sources include seafood (particularly shellfish), meat (especially beef), eggs, and dairy products [1]. Notably, vegetables lack TAU, except for red algae [1]. For instance, a species of red algae, “susabinori”, commonly consumed in Japan, contains approximately 1950 mg of TAU per 100 g of dry weight [2]. Energy drinks (e.g., Bacchus-D, Monster, Tab Energy, Red Bull, Rockstar) are other sources of TAU, containing 1 to 3 g per serving [3]. Additionally, TAU supplements are widely available in various forms, ranging from 500 to 2000 mg per serving [1]. Notably, the global market of TAU is increasing [4] and interestingly, women (77%) use more supplements than men (68%) [5].

TAU levels in vegans appear to be approximately half those of those consuming an omnivorous diet [6]. However, vegetarians and omnivores exhibit similar blood levels of TAU, likely due to the significant reduction in urinary excretion observed in vegetarians compared to omnivores [7]. Notably, low plasma or serum TAU levels have been associated with obesity, cardiometabolic risk, and type 2 diabetes mellitus (T2D) [8,9,10].

For many years, the sex and gender influence in medicine and health was neglected. Therefore, nutritional recommendations have not included the potential influence of sex and gender [11,12]. Unfortunately, most preclinical studies on TAU have been conducted on male rodents, which can synthesize TAU to meet their physiological requirements; obviously this fact partially decreases the translation value of results in humans. In addition, women have been neglected in clinical studies of TAU.

Although TAU has been the subject of extensive research, with over 1580 reviews listed in PubMed, most of them pay little or no attention to the influence of sex and gender on TAU’s biological activities. The primary aim of this review is to highlight how TAU activity is modulated by sex, from its synthesis to its clinical effects. Specifically, this review explores the potential beneficial roles of TAU in genetic and cardiovascular diseases (CVD), metabolic syndrome and diabetes mellitus, intrauterine growth restriction, pre-eclampsia, gestational diabetes mellitus, and developmental trajectories. Particular emphasis is placed on sex and gender differences, which are known to impact all of these conditions [13,14,15,16,17,18].

## 2. Search Strategy

A literature analysis for this narrative review was conducted using MEDLINE/PubMed and Google as primary research tools. Additional sources were identified through manual searches of other scientific databases and the reference lists of previous meta-analyses and reviews. The search focused on the following areas: taurine’s biological activity, sex and gender differences, and clinical studies involving taurine in cardiovascular, diabetic, and gestational conditions. The search strategy employed a combination of MeSH terms and related keywords for the specified topics, using Boolean operators “AND” and “OR” to refine the results. Two independent authors initially screened studies based on their abstracts. Selected articles were then reviewed in full for inclusion in the analysis.

## 3. TAU Endogenous Synthesis Is Influenced by Sexual Hormones

**Figure 1:**
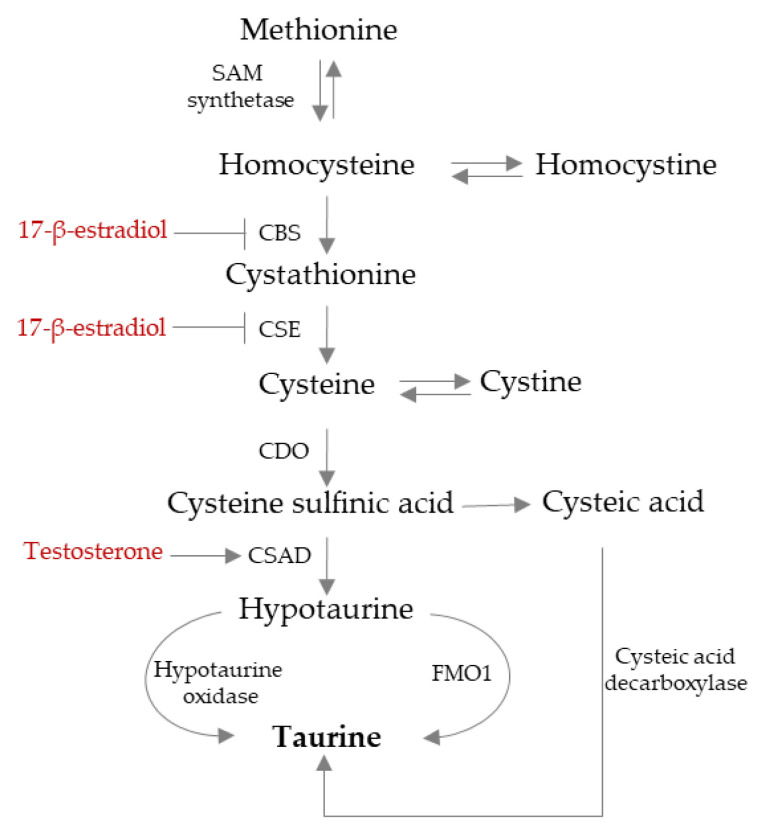
Effects of sexual hormones on endogenous TAU synthesis. Abbreviations: S-adenosyl-methionine (SAM); cystathionine β-synthase (CBS); cystathionine γ-lyase (CSE); cysteine dioxygenase (CDO); cysteine sulfinic acid decarboxylase (CSAD); monooxygenase subtype 1 (FMO1).

In adult humans (Figure 1), the liver synthesizes small amounts of TAU primarily via the “cysteine sulfinic pathway” [1]. More recently, a new enzyme, flavin-containing monooxygenase subtype 1, has been identified, and it converts hypotaurine to TAU [19]. In the human liver, flavin-containing monooxygenase subtype 1 is expressed during fetal development but decreases after birth and is absent in adults [19]. In mice, at least, the activities of flavin-containing monooxygenase subtype 1 and cysteine dioxygenase (CDO) isoforms are sex-divergent, being higher in females, which may result in approximately two-fold higher TAU tissue levels in females [19].

Sexual hormones impact TAU biosynthesis (Figure 1). Specifically, in mouse hepatic cells, testosterone upregulates the expression of the androgen receptor and cysteine sulfinic acid decarboxylase (CSAD), thereby promoting TAU synthesis. Vice versa, CDO is only minimally affected by testosterone [20]. All these testosterone-induced effects are counteracted by an androgen receptor antagonist [20]. Finally, bilateral orchidectomy reduces the expression of androgen receptors, CSAD, and liver TAU concentrations, and the injection of testosterone counteracts the effect of orchidectomy [20].

Conversely, in the liver of male mice, 17-β-estradiol does not impact CSAD expression and TAU synthesis [20]. In the liver of female mice, however, the expression of CSAD and CDO is linked to estrogen levels, with lower CSAD and CDO expression, as well as TAU levels, observed during estrus compared to diestrus [21]. In female mice, the administration of 17β-estradiol, both in vivo and in vitro, reduces TAU levels in serum and cultured cells by downregulating the expression of CSAD and CDO [21]. The action of 17β-estradiol appears to be mediated through the estrogen receptor-α, as this effect is partially inhibited by ICI-182,780, an antagonist of estrogen receptor-α [21]. Additionally, estrogens decrease the mRNA expression of cystathionine γ-lyase (CSE), which is involved in converting cystathionine to cysteine, while they increase the mRNA and protein expression of cystathionine β-synthase (CBS), facilitating the conversion of homocysteine to cystathionine in the myometrium of ovariectomized mice. In conclusion, sexual hormones influence TAU synthesis at least in some rodents [19]. The administration of methionine, a TAU precursor, elevates the plasma concentration of homocysteine more in females than in males in both humans and rats [22]. Interestingly, in wild Sprague Dawley rats, female livers express lower levels of glutamylcysteine ligase, the key enzyme for GSH synthesis, compared to male livers. Despite this, levels of L-methionine, glutathione, TAU, and malondialdehyde were found to be similar between sexes. However, L-cysteine levels were significantly higher, and hydrogen sulfide levels were lower in female rat livers [23].

Further, TAU synthesis in mammals is species-specific. For instance, cats have a limited capacity to synthesize TAU, while in rats, the enzymes involved in TAU synthesis exhibit higher activity [19]. On the contrary, rabbits, guinea pigs, and monkeys have a low level of TAU biosynthesis [19].

Finally, in both animals and humans, TAU biosynthesis declines with age [1]. Specifically, blood TAU levels in adults are approximately 80% lower than in children [24].

## 4. TAU Absorption and Distribution

TAU is actively absorbed in the ileum via the TAUT transporter (see below), and it is transported through the portal vein to the liver, where it is released into general circulation. TAU has a distribution volume of around 40 L [25]. A person with an average body weight of 70 kg (the average weight of a Caucasian man) may contain approximately 70 g of TAU, most of which is derived from the diet [26].

TAU levels are 100 times lower outside the cells than inside [27]. Table 1 summarizes variations in TAU levels in some biological fluids of healthy and diseased subjects.

In mammalian tissues, TAU is highly concentrated in the brain, retina, heart, skeletal muscle, and platelets, with especially high levels in leukocytes (20–50 mM) [45,46]. Overall, high intracellular TAU levels are found in tissues rich in mitochondria, whereas low levels are found in glycolytic tissues [1].

After TAU administration, plasma TAU levels typically peak within 1 to 2.5 h [47]. In healthy, overnight fasting adults, oral administration of 4 g of TAU increases blood levels from 30–60 μmol to approximately 500 μmol, with levels returning to baseline within 6.5 h [47].

## 5. TAU Elimination

TAU and TAU conjugates are excreted through urine and feces, respectively [27]. In the urine, the 70% is excreted as TAU and 25% as sulphate conjugates [48]. The kidney plays a crucial role in regulating TAU levels. Specifically, when TAU levels are elevated, the excess of TAU is eliminated in the urine. Conversely, when TAU levels are low, the kidney reduces its excretion and increases reabsorption [49]. Additionally, taurocholates are eliminated in the feces [49].

## 6. TAU Transporters: TAUT and Proton-Dependent Carrier (PAT1)

The TAU transporters were extensively reviewed by Baliou and coworkers [27]. Briefly, the concentration of TAU in the body is primarily regulated by its uptake through various ubiquitous TAU transporters [27]. The most extensively studied transporter is SLC6A6, which requires a specific ratio of sodium ions, chloride ions, and TAU (2:1:1) for optimal function [50]. Interestingly, in the gut, one paper shows that TAUT expression is similar in both men and women and is minimally affected by age [48,51]. The generation of TAUT knockout mice (TAUT-KO) underscores the critical role of TAU depletion in maintaining health, as these mice exhibit a great range of abnormalities, including a shortened lifespan, cardiomyopathy, mitochondrial alterations, diminished exercise capacity, and alterations of glucose homeostasis [1,7,52]. TAUT activity is downregulated by TAU via transcriptional and posttranscriptional mechanisms [27]. On the other hand, glucose, nitric oxide, hypertonicity, pro-oxidants, and inflammatory molecules can increase TAUT mRNA transcription [53]. In MCF-7 human breast adenocarcinoma cell lines, 17β-estradiol elevated TAU uptake by MCF-7 [54].

More complex is the situation in patients with DM. In peripheral blood mononuclear cells of people with T2D, the TAUT gene is overexpressed, but in patients with retinopathy, TAUT overexpression is abolished [34]. RNA-TAUT gene expression is also significantly upregulated in T1D patients, and it is related positively to HbA1c levels and inversely to plasma homocysteine. Finally, in T1D patients with retinopathy, RNA-TAUT upregulation seems to be blunted [35]. Finally, in humans, a small study suggests that intestinal TAU absorption is downregulated in individuals with T2D [27].

The less selective PAT1, which can also transport betaine, glycine, GABA, and proline [55], has been inhibited by the 17-α-ethinyl-estradiol and 17-β-estradiol in a non-competitive manner in Caco-2 cells and in vivo using Sprague Dawley rats. However, these estrogens do not inhibit PAT1 in oocytes [56]. In addition, in male Sprague Dawley rats, the administration of these hormones lowers the maximal plasma concentration of TAU and elevates the time maximus [56].

## 7. Biological Activities of TAU

**Figure 2:**
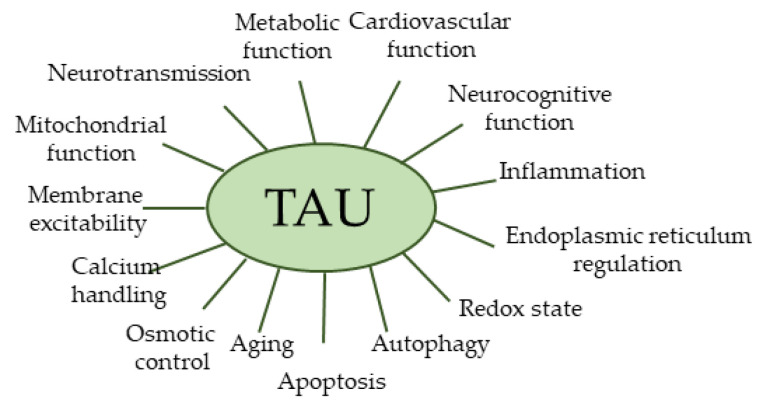
Biological activities of TAU.

Numerous reviews and papers indicate that TAU exerts pleiotropic physiological effects that are summarized in Figure 2 [1,2,24,47,48,49,57,58,59,60,61,62].

Briefly, TAU is a component of mitochondrial tRNAs and exists in two uridine conjugates, namely, 5-taurinomethyluridine and 5-taurinomethyl-2-thiouridine, which are linked to TAU’s antioxidant properties [47] that enhance mitochondrial protein synthesis [59]. Additionally, TAU enhances mitochondrial fatty acid oxidation and reduces MAPK activation [57]. Globally, TAU depletion leads to mitochondrial dysfunction and metabolic dysregulation in various tissues [47]. This, in turn, seems to involve insulin resistance leading to glucose metabolism impairment and T2D, as well as diabetic complications [63]. In aging, TAU seems to decrease cognitive decline [61,62]. In TAUT-KO 3-months-old male mice, heart mitochondrial complex I activity is diminished while oxidative stress is increased [60]. Beyond its mitochondrial effect, TAU plays a vital physiological role in endoplasmic reticulum stress, particularly in cardiac and skeletal muscle [49]. In hearts obtained from 12-month-old male mice, caspase-12 was found to contribute to cell death, indicating a pathological role of endoplasmic reticulum stress in aging mice deficient in TAU [60].

In numerous human and rodent cells, as well as different animal models, TAU reduces mitochondria and endoplasmic reticulum stress-dependent apoptosis [49,57,64] triggered by numerous stimuli such as oxidized low-density lipoproteins, drugs, toxicants [57,65], radiations [66], and caspase-12 (endoplasmic reticulum-localized enzyme).

In addition, TAU can influence autophagy—a cytoprotective process triggered by various factors but prolonged autophagy can lead to cell death [67]. In human hepatic stellate cell lines of unknown sex, TAU affects the expression of protein involved in autophagic process [67]. In particular, TAU downregulates the expression of p62 and upregulates the expression of key autophagic proteins such as LC3B, ATG5, and Beclin-1. In addition, transmission electron microscopy highlights ferroptosis features, an increase in autophagosomes, and a depression of the edge of nuclear membrane [68]. Furthermore, in matured 3T3-L1 mouse adipocytes of unknown sex and Leyding cells, TAU promotes autophagy [69,70]. However, in proximal tubular cells derived from human adult male kidneys (HK-2), TAU inhibits autophagy induced by oxidative stress or arsenic intoxication [71]. Whether these processes are sex and gender-dependent remains unknown, whereas in basal and irradiated male and female human umbilical vein endothelial cells, exposure to TAU does not modify autophagy [72]. Globally, the TAU effect on autophagy is not univocal and seems to depend on cell type and autophagic trigger.

TAU is an organic osmolyte involved in the regulation of cell volume [73]. Among its various physiological functions, TAU plays a crucial role in calcium homeostasis, particularly within the cardiovascular system. It contributes to excitation–contraction coupling by regulating calcium release from the sarcoplasmic reticulum and modulating myofibril sensitivity to calcium [74]. Additionally, TAU has the potential to lower blood pressure through multiple mechanisms [74].

TAU exhibits anti-inflammatory and antioxidant properties, which could potentially be due to reducing inducible nitric oxide synthase, cyclooxygenase-2, prostaglandin E2, NF-κB p65, and NF-κB DNA-binding in RAW 264.7 macrophages, a cell line derived from tumors of male mice, as well as in NR8383 cells, which are cloned alveolar macrophages from male rats exposed to liposaccharide [75,76]. Notably, TAU also exerts anti-inflammatory effects in the pancreas, helping to protect against β-cell destruction [77]. Additionally, TAU reacts with hypochlorous acid to form TAU-chloramine, which attenuates the effects of highly toxic hypochlorous acid [78]. The antioxidant and anti-inflammatory activities of TAU are further supported by a recent meta-analysis, which found that TAU supplementation can reduce levels of lipid peroxidation and C-reactive protein. However, it does not significantly impact the levels of other anti-inflammatory biomarkers such as tumor necrosis factor-α [79].

Some of the previously described actions of TAU may contribute to mitigating key hallmarks of aging, including mitochondrial dysfunction, cellular senescence, oxidative stress regulation, and inflammation [80,81].

However, it is important to note that these findings are derived almost exclusively from studies conducted on male animals, with limited consideration of sex-based biological differences and minimal inclusion of female subjects.

## 8. TAU and Cholesterol

The conjugation of bile acids is essential for maintaining cholesterol homeostasis [82], and the conversion of cholesterol to bile acids is facilitated by TAU in rodents and by glycine and TAU in humans [83]. This process is enhanced through the activation of cytochrome 450 enzyme (CYP) 7A1, which constitutes the rate-limiting steps in bile acids synthesis [83]. In aged male rats fed with a high-fat diet, TAU lowers serum cholesterol levels and increases CYP7A1 expression in hepatocytes. In farnesoid X receptor knockout (*Fxr*KO) mice, the supplementation with TAU either completely or partially restores bile acids and improves liver injury induced by hepatocellular carcinoma in *Fxr*KO female mice [84]. Additionally, in the liver, TAU upregulates organic anion transporting polypeptide 2 and betaine-homocysteine methyltransferase [85]. The hepatic bile acids formation is lower in females compared to males, and this sex difference seems to be due to a reduction of membrane lipid fluidity and to a selective reduction in sodium-dependent taurocholate peptide [86]. This last is more expressed in male rat liver than in female liver. Notably, estradiol decreases and increases its expression in males and in ovariectomized females, respectively [87], suggesting that estrogens are regulators of sodium-dependent taurocholate peptide activity. In addition, sex differences have been seen during aging [88]. Total serum bile acids were elevated at about 340% from 3 to 27 months only in female mice, whereas bile acids remained almost constant in male mice [88]. In female mice, it has been observed that the mRNA and proteins of hepatic bile acids uptake transporters, the Na(+)/taurocholate co-transporting polypeptide and the organic anion transporting polypeptide 1b2, decreased after 12 months, while the mRNA of CYP7A1, the rate-limiting enzyme for bile acids synthesis, increased from 3 to 9 months and remained high thereafter [89].

## 9. TAU Supplementation and/or Administration in Some Pathological Conditions

In clinical practice, TAU supplementation has been proposed as a complementary therapy for various conditions, including cancer, neurological, genetic, and other diseases [48,49]. As mentioned in the introduction, this review focused on genetic, CVD, and metabolic diseases. In clinical trials, TAU has been administered at much higher doses than the usual daily intake. However, there is a lack of large, high-quality clinical trials that establish a definitive role for TAU supplementation. In addition, the findings from animal and cellular studies have yet to be effectively translated into clinical practice [3]. Furthermore, the effects of sex and gender on TAU administration/supplementation have been minimally explored, despite the increasing recognition of the significance of sex and gender in various pathologies [90,91,92], because only a few female animals and only a few women have been recruited in clinical trials regarding cardiovascular or metabolic diseases.

## 10. Some Genetic Diseases

Human TAUT deficiency is caused by the homozygous variant Gly399Val of the TAUT SLC6A6 [93]. Children with this mutation exhibit nearly undetectable TAU levels and suffer from retinal degeneration and cardiomyopathy. TAU supplementation restores TAU levels, and after 24 months of treatment, a reduction in cardiac damage has been observed [93].

In Japan, the administration of TAU has been approved to treat MELAS (mitochondrial myopathy, encephalopathy, lactic acidosis, and stroke-like episodes), which in the late form is accompanied by DM and deafness as early symptoms [94]. Approximately 80% of patients have a mitochondrial DNA mutation that causes significant mitochondrial dysfunction [94], and TAU administration seems to ameliorate mitochondrial dysfunction and prevent stroke-like episodes in MELAS [95].

Oral TAU supplementation (75 mg/kg for 4 days), administered during a Phase I/II study in individuals with inherited CBS-deficient homocystinuria (eight men and six women, aged 8–50 years), did not affect markers of oxidative stress or inflammation. However, it improved brachial artery flow-mediated dilation in participants with baseline flow-mediated dilation values below 10% and in those with homocysteine levels greater than 125 μM [96].

## 11. TAU and CVD

Numerous biological actions of TAU may exert beneficial effects in CVD [97], encompassing membrane stabilization, calcium regulation, anti-inflammatory and antioxidant properties, antiaggregant activity, modulation of blood pressure (BP) and vascular tone, and hypocholesterolemic effects [97,98,99]. As already mentioned in the section “TAU and Cholesterol”, TAU may attenuate atherogenesis by decreasing the activity of 3-hydroxy-3-methylglutaryl-CoA reductase and increasing 7α-hydroxylase activity, thereby promoting cholesterol degradation. Additionally, TAU helps to lower reactive oxygen species, contributing further to its anti-atherogenic effects [97].

In addition, TAU supplementation (3 g/day for 4 weeks) in 22 women aged 33 to 54 years resulted in a significant increase in TAU levels and a modest but significant reduction in homocysteine levels by approximately 11% [100]. This potential reduction in hyperhomocysteinemia may contribute to the beneficial effects of TAU, as elevated homocysteine is a known risk factor for cardiovascular and metabolic diseases. It promotes the development of atherosclerosis through various mechanisms involving inflammation, alterations in lipoprotein metabolism, and oxidative stress [81].

Interestingly, the beneficial effect of TAU was also highlighted by an epidemiological study involving 16 different countries, which—despite significant differences among them—found a negative association between urinary TAU levels and ischemic disease [101]. In line with previous results, high levels of TAU have been associated with protection against cardiac ischemic diseases among hypercholesterolemic women [102].

In Japan, the authorization for TAU in the treatment of heart failure dates to 1985 [103], but it remains unlicensed in Europe and the United States [104]. In 2021, six trials (collectively, they enrolled 274 patients with heart failure—where treatment duration ranged between a few days and one year—five trials used the oral route, and one used the intravenous one) showed the beneficial effects of TAU in heart failure [25]. A subsequent systematic review identified 285 articles; however, only 11 met the inclusion criteria, and just 1 was of high quality. Despite these limitations, TAU supplementation improved exercise capacity, diastolic and systolic function, and hemodynamic parameters [104]. Finally, a meta-analysis (808 participants) concluded that TAU reduces heart rate, systolic BP (SBP), and DBP, improving the NYHA functional class, in a dose-dependent manner [97]. The effect of TAU on DBP has been observed only in hypertensive patients, while the decrease in SBP also involved healthy individuals, patients with heart failure, and those with other diseases [97].

The blood-vessel-relaxing effects of TAU have been described in numerous studies investigating its mechanisms of action on the vascular system, as recently reviewed [58,97]. Briefly, TAU may elevate availability of nitric oxide [97] and inhibit the renin–angiotensin–aldosterone system [97]. However, the precise effect on blood vessels at human levels is less clear. One randomized, double-blind, placebo-controlled study involving 120 prehypertensive individuals found that TAU supplementation significantly decreased ambulatory BP over 12 weeks, particularly in those with high–normal BP [105]. A successive meta-analysis including 103 patients of varying ages and health status who received TAU for 12 weeks evidenced that resting SBP and DBP were decreased by about 3 mm Hg in the TAU group [106]. Another meta-analysis that enrolled patients with liver or metabolic dysregulation such as DM and obesity showed that TAU decreased both SBP (4.67 mm Hg) and DBP (2.90 mm Hg), total cholesterol, and triglycerides [9,101].

The beneficial effects of supplementation of TAU in the CVD are, however, still unclear, and there is the urgent need for well-designed double-blind placebo-randomized clinical trials of good quality. It is important to underlie that, until now, women were under-represented in TAU cardiovascular trials (Table 2), even when numerous sex and gender differences were described in all CVD [107].

## 12. TAU in DM and Metabolic Syndrome

The pioneering study of Franconi and coworkers [10] showed that patients with T1D patients have lower plasmatic and platelets TAU concentrations that revert with TAU supplementation, which also reverts the anomalies observed in platelet aggregation. Insufficient TAU levels have been linked to diabetes, as confirmed by numerous studies that were reviewed by Ahmed et al. [126], and some of them are summarized in Table 2 and may be implicated in some DM complications [127]. In this regard, it is important to underline that in diabetic patients, there is a greater urinary excretion of TAU than intestinal absorption, which could result in lower TAU levels [126]. Although some small controlled clinical trials found inconsistent results about the efficacy of TAU supplementation in diabetic patients, a recent meta-analysis [127] including 209 T2D diabetic subjects—who were not stratified for sex—evidenced that TAU improved metabolic control but did not affect serum lipids, BP, or body composition. Interestingly, the antioxidant effects of TAU have been evidenced in T2D patients, whereas the importance of TAU anti-inflammatory action is less clear because only a few inflammatory biomarkers are decreased [128].

In T2D patients, TAU supplementation (3 g/day of TAU for 8 weeks) lowers blood fasting glucose, HbA1C, insulin, HOMA-IR (an index of insulin resistance), cholesterol, low-density lipoproteins, pentosidine, and methylglyoxal [121]. Further, the study of Premanath and colleagues [129] showed that 500 mg of TAU had beneficial effects in nephropathic T2D patients treated with angiotensin-converting enzyme inhibitors or angiotensin II type 1 receptor blockers and/or Clinidipin (a calcium channel blocker approved in China and Japan) plus 150 mg of N-acetyl cysteine. Notably, again, the data were not sex-stratified. In another study, TAU supplementation of diabetic women plus 8 weeks of total-body resistance exercise decreased HbA1c, lipids, and body fat percentage, improving insulin sensitivity, as expressed by the HOMA-IR [130]. Some authors even argued that TAU can be used as a complementary therapy to prevent DM and related complications [131,132]. In our opinion, well-designed clinical trials are needed, in view of the numerous sex and gender differences present in DM and its complications [90]. Notably, sex differences are present in experimental models of diabetes, although some of them do not translate in humans. For example, female animals are more resistant to diabetes induced by streptozotocin administration than male ones [133].

The protective effects of TAU against DM and its complications have been highlighted in various experimental models, supporting its proposed use in the management of DM and associated complications, including diabetic nephropathy, retinopathy, cataracts, neuropathy, and cardiomyopathy [127,134]. The possible TAU effect in DM depends on several mechanisms, which were reviewed by Ahmed et al. [126]. Briefly, numerous experimental studies indicate that TAU influences glucose homeostasis by modulating β-cell insulin secretion, enhancing peripheral insulin sensitivity, and interfering with insulin signaling through various mechanisms [132,135,136]. As previously mentioned, TAU’s ability to mitigate mitochondrial glucotoxicity [49] and its role in disrupting the link between endoplasmic reticulum stress, antioxidant activity, anti-inflammatory responses, and calcium regulation may contribute to its effects on DM and its complications. Indeed, all these mechanisms appear to play a significant role in the development of diabetic complications [49]. For instance, in preclinical studies, oxidative stress, inflammation, and the reduction in apoptosis are key factors in the progression of diabetic nephropathy [137] and retinopathy [138]. In Sprague Dawley rats fed with a high-fat and high-glucose diet, TAU reduced islet pathological changes and improved peripheral insulin sensitivity, as assessed by HOMA-IR [139]. In DM, the TAU effect on osmoregulation could be relevant, because hyperglycemia leads to cellular osmotic stress [140].

Diabetic retinopathy and age-related retinal degeneration are among the major causes of blindness worldwide [45]. In addition, low plasma TAU has also been found in Leber hereditary optic neuropathy [141] and in central serous chorioretinopathy [45]. Interestingly, low TAU plasma levels are associated with the development of retinal degeneration in people with diabetes [142,143]. Conversely, a small study showed that the elevation of plasma TAU may prevent vision loss in patients with T2D [143]. The administration of TAU in the prevention of retinal degeneration has recently been reviewed [45]. Briefly, TAU is needed for retinal development and health, as also demonstrated by retinal degeneration shown in knockout animals for the TAU transporter [144] and in humans with genetic alteration of the TAU transporter [93]. The activity of TAU depends on neuroprotection, antioxidant, and anti-inflammatory activities; the reduction in retinal neovascularization; pigment epithelium; phagocytosis; and antiapoptotic activities [45,145]. In fact, numerous sources of animal data indicate that dietary TAU supplementation prevents glial alterations in the retina of diabetic rats [146]. TAU supplementation reduces oxidative stress and alters Na^+^ K^+^ ATPase in the retina of male rats with streptozotocin-induced diabetes, being, for this purpose, more effective than vitamin E [147,148]. A recent paper evidenced that TAU administration may protect retinal cells in streptozotocin-induced diabetic rats activating TAUT lowering retinal apoptosis and reactive gliosis and ameliorating retinal synaptic connections [149].

Some influences of sex on the effect of TAU in experimental diabetic settings or clinical studies are reported in Table 3. Considering that DM is a disease that presents numerous sex and gender differences [90,150,151], studies should be done according to a sex and gender perspective.

A very recent meta-analysis (1024 participants from 25 randomized controlled trials) evidenced that oral TAU exerts a protective effect on metabolic syndrome, decreasing the risk for CVD and T2D, as well as decreasing SBP and DBP, total cholesterol, and fasting glucose [97]. These data suggest that TAU supplementation can be used in metabolic syndrome conditions. However, the meta-analysis does not indicate, with some rare exceptions, any difference according to the sex of participants.

## 13. TAU in Fetal and Neonatal Life: Intrauterine Growth Restriction, Pre-Eclampsia, Gestational Diabetes, and Developmental Trajectory

Mammalian fetuses and infants require the acquisition of this amino acid from maternal blood or breast milk [53], and TAU levels are higher in umbilical vein plasma and amniotic fluid than in maternal blood [53]. Higher TAU intake is positively correlated with the weight and height of infants [163]. Pregnancy complications are risk factors for both CVD and DM in adults [164,165]. Importantly, the placental levels of TAU in infants with a low birth weight and/or in small infants for gestational age are lower than those found in the placentas of neonates of normal birth weight [166] and of neonates fed with formula milk supplemented with TAU [167]. Because of this, TAU has been added to commercial infant formula and long-term parenteral and amino acid solutions [53,168,169]. Importantly, the placenta transfer of TAU from mother to offspring mainly occur through TAUT and via breast milk. Both transfers are regulated by numerous factors such as maternal obesity, pre-eclampsia, and malnutrition [53]. Pre-eclampsia, obesity, and high glucose levels are associated with reduced TAUT activity [53] and induce impairment of fetal growth, with the latter being associated with undesirable outcomes such as obesity, T2D, and an increase in risk of CVD later in adult life [170,171,172].

### 13.1. TAU and Developmental Trajectories

As mentioned above, it has been suggested that during prenatal and neonatal life, TAU may influence organ development, fetal growth, and developmental trajectory [53], being crucial for retinal development and the health of many other organs [45,173,174,175,176]. According to the hypothesis linking developmental programming with the risk of metabolic and CVD in adult life [172], much evidence suggests that perinatal TAU may program adult function through different mechanisms including epigenetic ones [177]. Perinatal TAU prevents hypertension in SHR spontaneously hypertensive rats [178], while TAU deprivation during fetal growth increases the risk of T2D in postnatal life [179]. Perinatal TAU supplementation increases the mean arterial pressure only in adult male rats whereas amino acid depletion increases arterial pressure only in adult female rats [176]. Furthermore, both TAU depletion or TAU supplementation in early life change renal function and autonomic nervous control of BP [175,176]. In mice, neonatal TAU administration decreases basal glucose levels and the area under the curve after glucose loading in adult males [180]. Thus, in rodents, neonatally administered taurine produces enduring effects in a way that could be advantageous for the control of glucose homoeostasis. Globally these data suggest a sex–adult effect of perinatal TAU on arterial pressure control. The TAU protective effect in offspring born from diabetic mothers also involves cellular fate, because the hepatic autophagy and apoptosis induced by maternal diabetes are reduced by TAU administration in streptozotocin-treated rats [181]. In male mice only, the daily exposure to a brief maternal separation plus intraperitoneal injection of physiological solution induced an increase in body weight, glycemia, insulin, and triglycerides, where all these effects were reduced by neonatal TAU administration [182], suggesting that TAU supplementation could protect from obesity, DM, and CVD at least in males [183]. In the same experimental model, 30-day-old mice showed higher pain in hot plate and in tail-flick tests. Interestingly, the hyperalgesia observed in the hot plate was reversed by postnatal TAU administration [184]. A further confirmation of TAU importance in early life involves the development of the neocortex; in fact, TAU tonically activates GABAA and glycine receptors and regulates Cl^−^ homeostasis, affecting pivotal developmental processes [185].

### 13.2. Intrauterine Growth Restriction (IUGR)

In experimental models (rat), intrauterine growth restriction reduced by about 34% the sodium-dependent uptake of TAU, while sodium-independent uptake was scarcely influenced [53]. Additionally, gestational protein restriction modified gene expression in the liver and skeletal muscle, which was partially rescued by TAU administration [53].

### 13.3. Pre-Eclampsia

In experimental pre-eclampsia, TAU administration between the 16th to the 19th day of rat pregnancy led to normalization of the metabolic processes such as the level of malondialdehyde, nitric oxide, lactate, and lactate dehydrogenase activity [186]. In rats, the activity of TAUT that transports TAU from maternal blood to syncytiotrophoblasts was reduced in pre-eclampsia [187]. In a rat ADMA-like model of pre-eclampsia, the hemostasis alterations are reduced by TAU administration [188]. Clinically, in pregnancy, serum TAU levels were decreased in first-trimester pregnant women who subsequently developed pre-eclampsia [189]. This TAU decrease appeared to be the most discriminative biomarker, in combination with mean BP, in predicting pre-eclampsia when measured in serum during the first-trimester of pregnancy [189].

### 13.4. Gestational Diabetes Mellitus

This common pregnancy complication of gestational diabetes mellitus affects both fetuses and mothers. Mothers can develop gestational hypertension, pre-eclampsia, and risk of developing T2D or CVD [190], while the children may have adverse effects such as macrosomia, shoulder dystocia or birth injury, neonatal hypoglycemia, and higher risk of cardiometabolic diseases later in life [190]. Interestingly, in non-obese diabetic mice, perinatal TAU delayed the onset of DM from 18 to 30 weeks in female offspring and from 30 to 38 weeks in male offspring [183]. In addition, in a streptozotocin rat model of gestational diabetes mellitus, the maternal administration of TAU reduced edema, apoptosis, and autophagosome in offspring in the liver [181]. A Chinese study involving 398 multiparous pregnant women found that high TAU levels in women were associated with a reduced risk of gestational diabetes mellitus as well as with an increase in HOMA-β index, suggesting an improvement in insulin secretion [191]. Another Chinese study showed that in the first trimester of pregnancy, serum TAU levels were negatively related to the risk of gestational diabetes mellitus; however, this association disappeared in the second and third trimesters [183]. Furthermore, elevated maternal plasma TAU levels were linked with increased insulin secretion, as measured by the HOMA-β index [41]. A small study reported that plasma TAU was lower in women who had experienced gestational diabetes mellitus about six years earlier, being negatively associated with the area under the curve of glucose measured during pregnancy and positively associated with indices of glucose-dependent insulin secretion such as post-gestational and gestational C-peptide/fasting plasma glucose [42]. Therefore, lower plasma TAU may be tracked with impaired glucose metabolism [42].

## 14. The Safety Profile of TAU in Humans

Notably, TAU seems to have a good safety profile, which is important because it is widely used in supplements, energy and functional drinks, and infant formula [192,193]. However, the excessive consumption of TAU if used as a dietary supplement may induce some potential side effects such as nausea, vomiting, diarrhea, and neurological symptoms (dizziness, tremors, and headache) [194,195]. Nonetheless, a recent meta-analysis has not been able to identify significant adverse effects during chronic TAU treatment compared to controls [97]. A risk assessment study established the upper level of TAU supplementation is 3 g per day [196] and the European Food Safety Authority indicates the safe limit at 6 g/person per day [197].

However, a recent paper published in Nature raises concerns about the safety of high doses of TAU, as it shows that leukemia cells can uptake TAU, which in turn enhances glycolysis and promotes cancer growth [198]. Since TAU is widely used as a supplement, including for mitigating the side effects of chemotherapy [180], the findings of Sharma’s paper suggest caution in using supplemental TAU in patients with leukemia.

To our knowledge, no sex and gender-oriented studies have been conducted to identify potential adverse effects of TAU. However, it is important to note that TAU inhibits CYP2E1, an enzyme that is more highly expressed in men than in women [199]. Therefore, the metabolism of drugs that are substrates of this enzyme may be affected by TAU coadministration in a sex-dependent manner [3].

## 15. Is TAU Activity Influenced by Sex and Gender?

The impact of sex on TAU action has been minimally studied. However, sporadic findings suggest that TAU activity may be influenced by sex and gender. For example, as mentioned in the sections “TAU biosynthesis” and “Taurine and Cholesterol”, sexual hormones play a role in TAU endogenous synthesis, and the formation of bile salts is different according to sex.

In addition, in mice brains, total TAU levels are more elevated in males than females [155], and TAU demonstrates neuroprotective effects against seizures induced by pilocarpine solely in male rats [200]. Moreover, some researchers argue that the antiaging effect of TAU is more pronounced in females than in males [24].

Some sex differences have also been seen in humans. In the placenta, the target mRNA levels of the amino acid transporter 1 (*LAT1*) and of *SLC6A6* (which encodes TAUT) are generally higher in the mother carrying a male fetus [201], as occurs for TAU levels in male human umbilical veins cells and cord blood [201].

Differently from rodents, TAU levels in the cerebrospinal fluid are higher in women than in matched men [29]. In human traumatic brain injury, TAU decrease is more pronounced in men than in women [202]. Recent evidence indicates that in adult women, TAU helps to prevent age-related decreases in superoxide dismutase levels [203]. Notably, earlier studies showed that in obese women, TAU decreased BP, hypercholesterolemia, body mass index, and inflammation. The additional sex differences outlined in the previous sections collectively underscore the importance of tailoring TAU supplementation and administration to account for sex-related similarities and differences. Furthermore, the prevention and management of non-communicable diseases (e.g., CVD and DM) are complex, necessitating long-term studies. Such research should prioritize the appropriate inclusion of women and the stratification of results by sex to ensure comprehensive and equitable outcomes.

## 16. Conclusions and Future Perspectives

In our opinion, there is an important question regarding the transferability of the data obtained in experimental animals, with exceptions such as guinea pigs, rabbits, and monkeys, to humans. In fact, mice and rats synthesize TAU in quantities sufficient for their needs, while this is not the case with humans. Probably, it is the moment to change species, utilizing guinea pigs, rabbits, and monkeys that have low levels of TAU biosynthesis comparable to humans [20]. Despite the preclinical results (mainly performed in male mice and rats), the mechanisms underlying the potential beneficial effects of TAU need to be further elucidated both in males and females, but the data come from small, poorly designed studies, so they should be taken with caution.

Adult CVD and DM are often influenced by early life events. In contrast, TAU administration during the perinatal period or in adulthood has the potential to reduce the risk of both CVD and DM. However, perinatal TAU depletion may contribute to the development of these conditions through multiple mechanisms, although the exact pathways remain unclear. In adults, TAU supplementation appears to lower SBP and DBP, improve NYHA classification, and enhance outcomes in heart failure and overall cardiovascular health. These findings, however, are primarily based on small and methodologically limited studies and should therefore be interpreted with caution. Indeed, many of the conditions for which TAU supplementation has been proposed exhibit significant sex and gender differences in prevalence, diagnosis, outcomes, and treatments [204]. For example, women tend to experience higher mortality rates and poorer prognoses following cardiovascular events [92]. Despite this, clinical and preclinical studies have predominantly focused on men and male animals. In other words, research on TAU has largely overlooked the female sex and gender, except in the context of pregnancy and lactation, so undermining its scientific rigor and generalizability. The inclusion of females in research is mandated by numerous organizations [205]. This is particularly important given the role of sex hormones in the endogenous synthesis of amino acids and the interaction between cholesterol and TAU. TAU also plays a significant role during pregnancy, showing benefits in conditions such as pre-eclampsia, gestational diabetes mellitus, and intrauterine growth restriction.

Another important aspect is the beneficial effect of TAU in aging. The relationship between TAU and longevity has been discussed in various parts of this manuscript. Although TAU levels tend to decline with age, it is challenging to clearly demonstrate the effect of aging on whole-body TAU content. This decline may vary depending on the animal species and experimental conditions. In humans, however, lower TAU levels are associated with several age-related diseases. Interestingly, the antiaging effects of TAU appear to be more pronounced in females. Future studies are needed to clarify the relationship between dietary TAU intake and healthy life expectancy.

Importantly, TAU appears to be safe, although this aspect still requires more investigations. Finally, high-quality preclinical and clinical investigations should include both sexes to facilitate the supplementation and administration of TAU in clinical settings to prove the beneficial effects of this amino acid.

## Figures and Tables

**Table 1:** TAU levels in some biological fluids of men and women.

Number and Sex of Subjects	Age (Years)	Ethnicity	Biological Fluid	Healthy Subjects	Disease	TAU Levels	References
26 cases and 26 controls; 46% women	>60	Not specified	Aqueous humor	26 controls	primary open-angle glaucoma	<in glaucoma	[28]
21 men 71 women	55.9 ± 18.1 44.8 ± 15.9	Chinese	CSF	cognitively healthy	Not specified	=	[29]
12 men 20 women	65	Swiss	CSF	cognitively healthy	No disease	>women	[30]
15 schizophrenic women 11 schizophrenic men 9 healthy women 6 health men	21–53	German	CSF	15 controls	schizophrenic disorders	<patients =in men and women	[31]
83 healthy men 85 healthy women	18–40	Italian	Plasma	85 controls	No disease	=	[32]
16 (6 women)	18–65	Lebanese	Plasma and urine	8 controls	6 T2D + 2 T1D	Basal TAU = After an oral TAU load, DM patients < plasma TAU levels at peak	[33]
81 cases and 45 controls; 24% women	>40	Italian	Plasma	45 controls	T2D	<in T2D =in men and women	[34]
35 cases and 33 controls; 56% women	>40	Italian	Plasma	33 controls	T1D	=in cases and controls	[35]
59 cases and 28 controls; 54% women	37–82	Turkish	Plasma	28 controls	T2D	<TAU especially in patients with neuropathy	[36]
39 cases and 34 controls	35–70	Italian	Plasma	34 controls	T1D	<plasma TAU in patients	[10]
89 cases and 28 controls; 44% women	>50	Multiethnic	Plasma	28 controls	COVID-19	>TAU in cases	[37]
93 psoriasis vulgaris; 20 palmoplantar pustulosis; 20 controls; 34% of women	>18	Korean	Plasma	20 controls	Psoriasis vulgaris and Palmoplantar pustulosis	>TAU in psoriasis vulgaris =TAU in palmoplantar pustulosis	[38]
	23–88	Japanese	Serum	controls		M < with age F no change with age	[39]
486 women 259 with GDM	GDM age 29.2 ± 3.3 Control age 29.2 ± 2.7	Chinese	Serum	227 controls	GDM	<TAU is associated with the risk of GDM	[40]
47 cases 47 controls	33 32	Chinese	Serum	47 controls	GMD	TAU < 1 trimester TAU = 2 trimester	[41]
43 cases; 7 IGT; 22 controls; GDM	32 ± 3 34 ± 3 34 ± 4	Italian	Serum	22 controls	GDM	<TAU in cases	[42]
72 cases and 401 controls; 44% women	40–65	Chinese	Serum	401 controls	MetS	<TAU in cases	[43]
62 with GDM 77 controls	34.27 ± 5.07 32.69 ± 4.72	Greek	Serum	77 healthy control	GDM	<TAU in cases	[44]

CSF—cerebrospinal fluid; IGT—impaired glucose tolerance; MetS—metabolic syndrome; GDM—gestational diabetes mellitus.

**Table 2:** Clinical trials of TAU in CVD and DM.

Disease	Number of Patients	Ratio f/m	Dose (g) and Duration	Age (Years)	Geographical Localization	References
Healthy men	29	0/29	6/day; 15 days	TAU 25.4 ± 1.0 Placebo 25.2 ± 1	Japan	[108]
Congestive HF	58	30/28	6/day; 4 weeks *	38–89	Japan	[109]
Congestive HF	14	5/9	6/day; 4 weeks *	68.71 ± 9.10	Japan	[110]
Congestive HF Class II–III	55	NR	1/day, 1 month	45–62	Russia	[111]
HF with LVEF < 50%	29	3/26	1.5/day; 2 weeks	60 ± 6	Iran	[112]
Congestive HF Class II–III	40	NR	1.5/day; 3 months	40–70	Russia	[113]
Idiopathic dilated cardiomyopathy	17	6/11	3/day; 6 weeks	NA	Japan	[114]
Prehypertensive individuals	86	44/42	1.6/day; 12 weeks	56.75 ± 8.26	China	[105]
Borderline hypertension	19	NR	6/day; 1 week	20–25	Japan	[115]
Aorto-coronary bypass	38	2/36	3/day; 35 days	TAU 62 ± 11 Placebo 69 ± 5	Canada	[116]
Coronary heart disease/Heart valve defects	48	12/36	0.5/day; 3 months	TAU 49.79 ± 1.4 Placebo 48.65 ± 1.5	Russia	[117]
Functional Capacity, Myocardial Oxygen Consumption, and Electrical Activity	16	NR	0.5/day; 2 weeks	TAU 62.12 ± 12 Placebo 60.13 ± 8.3	Iran	[118]
MetS and diastolic HF	78	59/19	1/day 12 months	31–66	Russia	[119]
HF	16	NR	1.5/day 2 weeks	TAU 61.7 ± 6.4 Placebo 60.4 ± 6.9	Iran	[120]
T2D	46	32/14	3/day	TAU 42.74 ± 7.21 Placebo 43.52 ± 6.94	Canada	[121]
T2D	120	97/23	3 g/day; 8 weeks	TAU 52.13 ± 8.1 Placebo 53.13 ± 8.1	Iran	[122]
T2D	18	0/18	1.5/day; 8 weeks	40 ± 8	Denmark	[123]
T1D	19	0/19	1.5/day; 2 weeks	28.0 ± 2	Ireland	[124]
Patients with hepatic venous pressure gradient ≥ 12 mm Hg	22	8/14	6/day; 4 weeks	52 ± 11	Austria	[125]

Modified from [97], * cross-over study 4 weeks with TAU. Heart failure—HF; Metabolic syndrome—MetS; not reported—NR.

**Table 3:** Some sex differences in different experimental DM including gestational diabetes mellitus.

Animals	DM Models	Sex	Effects	References
Mice	High-Fat Diet + STZ	Males	TAU induces inulin metabolism	[152]
Mice db/db and C57BL6/KsJ mice (control)	Genetic	Males	*Dendrobium officinale* ameliorates the liver metabolism pathway of TAU	[153]
db/db mice (T2D) compared to wild-type (WT) C57Bl6/J mice	Genetic	Males and Females	TAU > only in the female brain	[154]
Wild-type mice with C57BL/6		Males and Females	Brain TAU > only in males	[155]
C57BL/6 mice	STZ	Males	Human amniotic mesenchymal stem cells promote the increase in TAU in intestinal cells	[156]
Zucker rat	Genetic	Males	Plasma TAU<	[157]
Sprague Dawley rats	STZ	Males and females	Glycemia, mechanical allodynia, >females, Weight loss > males Aromatase > at 12 weeks in females	[158]
Sprague Dawley rats	STZ	Males	In the liver, TAU increases antioxidant biomarkers and reduces inflammation	[159]
Sprague Dawley rats	STZ	Sex of animals unreported	TAU protects from diabetic neuropathy	
Type 2 diabetic Goto–Kakizaki rats	Genetic	Males and Females	Sexually dimorphism in nerve repair in diabetic rats; axonal outgrowth after transection and activated Schwann cells > in males than in females	[158]
Hamster	Hereditary cardiomyopathy females present more lesions	Males and Females	Short-term TAU prevents the development of cardiac hypertrophy only in males	[160]
Hamster	Hereditary cardiomyopathy females present more lesions	Males and Females	Long-term TAU prevents the development of hypertrophy in both males and females	[161]
Human adipocyte and CCL-241 cells from obese patients	Obesity	Men and Women	NLRP6 is upregulated by TAU in CCL-241 enterocytes	[162]
Human	GDM	Women	Low serum level of TAU is a risk factor for GDM	[40]
Human	GDM	Women	TAU reduced in women with a history of prior GDM	[42]

NOD-like receptor family pyrin domain containing—6-NLRP6; streptozotocin—STZ; gestational diabetes mellitus—GDM.

## Abbreviations

The following abbreviations are used in this manuscript:

Blood pressure: BP. Cardiovascular disease: CVD. Cerebrospinal fluid: CSF. Cystathionine β-synthase: CBS. Cystathionine γ-lyase: CSE. Cysteine dioxygenase: CDO. Cysteine sulfinic acid decarboxylase: CSAD. Diabetes mellitus: DM. Diastolic BP: DBP. Heart failure: HF. Metabolic syndrome: MetS. Proton-dependent carrier: PAT1. Systolic BP: SBP. TAU transporter: TAUT. Taurine: TAU. TAUT knockout mice: TAUT-KO. Type 2 diabetes mellitus: T2D.
